# Establishing the Minimal Clinically Important Differences for Sagittal Hip Range of Motion in Chronic Stroke Patients

**DOI:** 10.3389/fneur.2021.700190

**Published:** 2021-09-01

**Authors:** Agnieszka Guzik, Mariusz Drużbicki, Lidia Perenc, Andżelina Wolan-Nieroda, Andrea Turolla, Paweł Kiper

**Affiliations:** ^1^Department of Physiotherapy, Institute of Health Sciences, Medical College, University of Rzeszów, Rzeszów, Poland; ^2^Laboratory of Kinematics and Robotics IRCCS San Camillo Hospital, Venice, Italy; ^3^Azienda Unità Locale Socio Sanitaria 3 Serenissima Physical Medicine and Rehabilitation Unit, Venice, Italy

**Keywords:** chronic stroke, minimal clinically important difference, hip, range of motion, gait

## Abstract

Many researchers have pointed out that decreased sagittal range of motion (ROM) in the affected hip joint is a common consequence of stroke, and it adversely affects walking performance and walking speed. Nevertheless, the minimal clinically important differences (MCID) in hip-related kinematic gait parameters post-stroke have not yet been determined. The present study aimed to define MCID values for hip ROM in the sagittal plane i.e., flexion–extension (FE), for the affected and unaffected sides at a chronic stage post-stroke. Fifty participants with hemiparesis due to stroke were enrolled for the study. Four statistical methods were used to calculate MCID. According to the anchor-based approach, the mean change in hip FE ROM achieved by the MCID group on the affected/unaffected side amounted to 5.81°/2.86° (the first MCID estimate). The distribution-based analyses established that the standard error of measurement in the no-change group amounted to 1.56°/1.04° (the second MCID estimate). Measurements based on the third method established that a change of 4.09°/0.61° in the hip ROM corresponded to a 1.85-point change in the Barthel Index. The optimum cutoff value, based on ROC curve analysis, corresponded to 2.9/2.6° of change in the hip sagittal ROM for the affected/unaffected side (the fourth MCID estimate). To our knowledge, this is the first study to use a comprehensive set of statistical methods to determine the MCID for hip sagittal ROM for the affected and unaffected sides at a chronic stage post-stroke. According to our findings, the MCID of the hip FE ROM for the affected side amounts to 5.81° and for the unaffected side to 2.86°, in patients with chronic stroke. This indicator is extremely important because it allows clinical practitioners to assess the effects of interventions administered to patients, and to interpret the significance of improvements in sagittal kinematic parameters of the hip; ultimately, it may facilitate the process of designing effective gait reeducation programs.

## Introduction

Stroke is the second most common cause of mortality worldwide and the third most frequent cause of disability and premature death (1). Stroke is also the leading cause of disability in adults in Poland, where annually around 70,000 people suffer the first stroke and around 30,000 recurrent strokes are observed (2). Lower-extremity function is commonly impaired after stroke, and it is assumed that more than one in two individuals who are able to walk unassisted at a chronic stage of recovery after stroke are found with kinematic and spatiotemporal asymmetry of gait pattern (3, 4). Impaired gait after stroke leads to the most severe sense of loss since the patient's return to the society and to their workplace largely depends on the improvement and recovery of walking ability (5). Therefore, rehabilitation programs designed for stroke patients primarily focus on gait reeducation and numerous researchers for decades have investigated hemiplegic gait in the attempt to develop efficient approaches to gait analysis (6).

The importance of gait kinematics assessment was emphasized by Boudarham et al. (3). As established by other researchers, accurate evaluation of gait kinematics enables assessment of improvement in functional abilities achieved by the patient; it is helpful in predicting the outcomes, in planning therapy and monitoring effects of the post-stroke rehabilitation (7–9). Although observational gait analysis is the most common approach to evaluating gait kinematics, there are a limited number of diagnostic tools designed specifically for assessing gait patterns in patients with hemiparesis after stroke (8, 10). Given the above, three-dimensional gait analysis (3DGA) today is a golden standard in this area as it provides objective data related to kinematic parameters (11–13).

It has been established that 3DGA kinematic indices are most reliable in assessing the hip and knee in the sagittal plane (12, 14). Conversely, the lowest reliability and the highest number of errors were reported in measurements related to the hip and knee in the transverse plane (12). Furthermore, the measure referred to as the minimal clinically important difference (MCID) was introduced as a central concept in research focusing on clinical measures post-stroke (15). MCID is defined as “the smallest difference in score in the domain of interest which patients perceive as beneficial and which would mandate (.) a change in the patient's management” (16). So far, MCID has been calculated for only one of the above major 3DGA kinematic predictors of walking performance post-stroke, i.e., for knee (17). In addition to that, some related studies have aimed to determine the minimal detectable change (MDC) in individuals post-stroke (18, 19). For instance, Keser et al. (18) calculated MDCs for kinematic gait variables during treadmill walking, for which they used data from repeated testing sessions in individuals with post-stroke gait impairments. Furthermore, Geiger et al. (19) established the MDC for hip, knee, and ankle angles in the sagittal plane during both stance and swing phases in patients with chronic stroke. According to comparative definitions offered by Wu et al. (20), the MDC is the smallest change in scores, not resulting from measurement error, possibly reflecting “true change,” whereas the MCID is understood as the smallest change in scores, seen as clinically meaningful and corresponding to a significant beneficial change in patient's self-assessed health status. Hence, the subjective improvement is not taken into consideration by MDC, while this factor is accounted for by MCID, and this is the added value of the latter concept, compared to the former, and at the same time the added value of our article compared to the above publications. This study is the second part of a broader research project. The first part aimed to estimate the MCID for knee range of motion (ROM) in the sagittal plane for the affected and unaffected side at a chronic stage post-stroke (17). Taking into consideration the aforementioned facts, we have decided to continue the related research, this time focusing on the kinematic parameter of hip sagittal ROM, in line with the evidence suggesting the high reliability of 3DGA (12). We were also motivated to carry out this research by the fact that many researchers have pointed out that decreased sagittal ROM in the affected hip joint is a common consequence of stroke, significantly contributing to impaired walking performance and walking speed (21–26). Moreover, hip sagittal ROM plays an important role in obstacle crossing. This operation involves larger movements which have to be performed by the hip joint in order to move the body not only horizontally, but also vertically; therefore, it may be particularly difficult for hemiplegic patients since their hip sagittal ROM is severely limited (5, 27).

From clinical practitioners' viewpoint, the MCID is highly useful since it enables the evaluation of effects achieved by patients receiving therapy; it may also provide effective support in the process of designing treatment strategies (17, 28). Given the above, the current study aimed to establish MCID values for hip ROM in the sagittal plane, i.e., flexion–extension (FE) for the affected and unaffected sides in patients with chronic stroke. The study estimated MCID values using a patient anchor-based method, distribution-based method, linear regression analysis, and specification of ROC curve.

## Methods

### Participants

**Figure 1:**
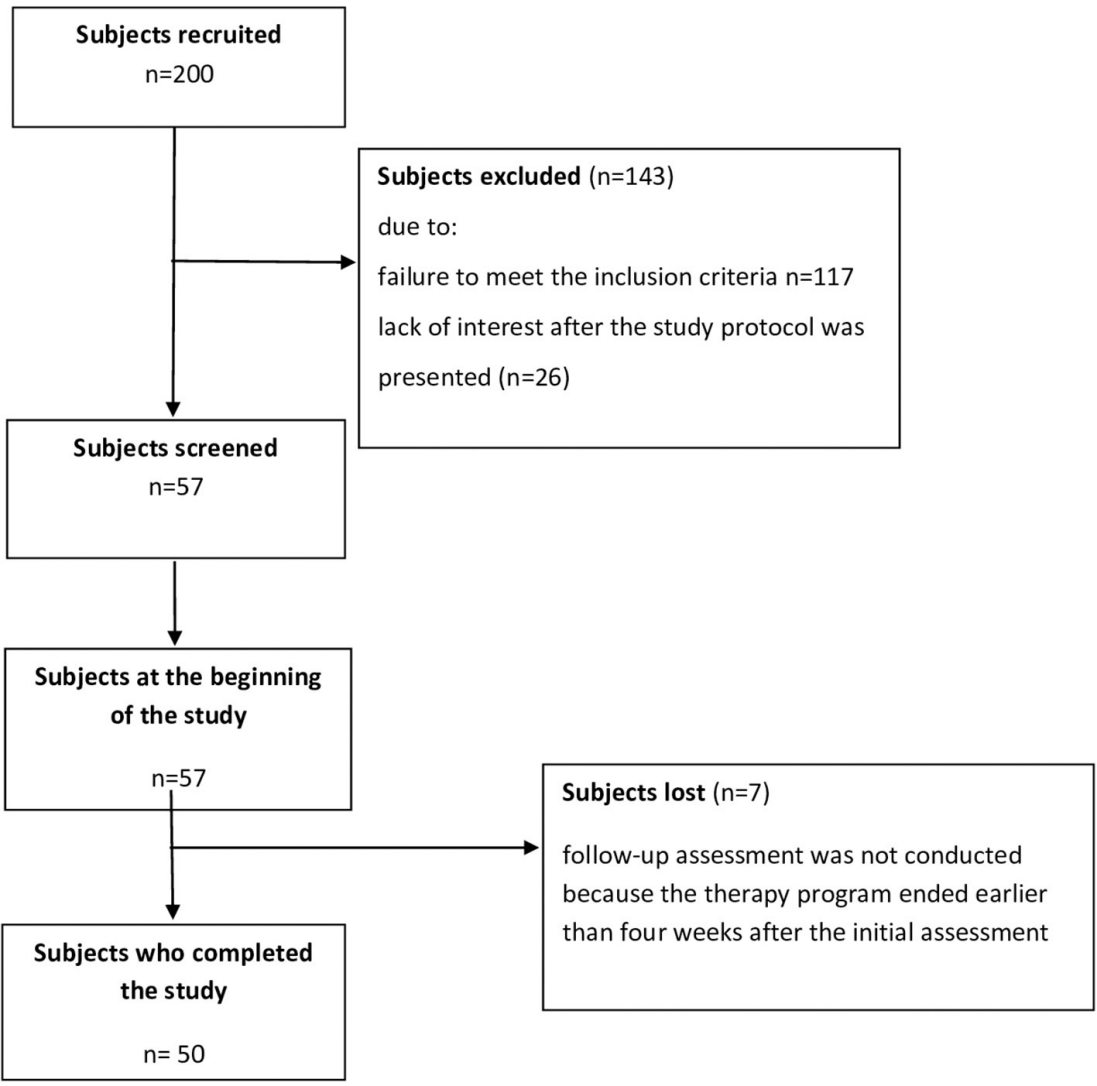
Flow diagram relative to the enrolment of subjects.

Fifty individuals after a stroke in a chronic phase of recovery (mean age 60.9 ± 11.2 years; mean time from stroke 42 months, ranging from 8 to 120 months; 18 females, 32 males; 15 patients with right hemisphere lesions, 35 patients with left hemisphere lesions) were identified in a database of a rehabilitation clinic. The following eligibility criteria were specified for those recruited for the study: age in the range of 30–75 years; single ischemic stroke experienced; a minimum of 6 months from the stroke incident; unilateral paresis; Brunnström's motor recovery stages 3–4; and a score of 3 or more in the Functional Ambulation Category walking test, including an ability to get up and walk a minimum distance of 10 m without support from another person (ambulatory assistive devices permitted, gait velocity > 0.4 m/s). Exclusion criteria were defined as follows: recurrent stroke event(s) experienced, unstable medical condition, orthopedic impairments in the lower extremities, inflammation and/or pain in the musculoskeletal system adversely affecting gait and/or associated with a need to take anti-inflammatory drugs, and inability to grasp the instructions and to perform required tasks due to cognitive disorders. The study protocol was approved by the local Bioethics Commission at University of Rzeszow's Medical Faculty. Compliance with the Declaration of Helsinki was ensured. Informed consent was given in writing by all the study participants. No adverse events occurred during the study procedures. The demographic and clinical characteristics of the study participants are presented in Table 1. The flow diagram relative to the enrollment of subjects is presented in Figure 1.

**Table 1:** Demographic and clinical information about the study participants.

**Variable (*N* = 50)**	***N***
Sex (women/men)	18/32
Hemisphere lesions (right/left)	15/35
Brunnström's motor recovery stages 3/4	29/31
Functional Ambulation Category 3/4/5	19/22/9
Spasticity in the modified Ashworth scale 1/1+	15/35
	**Mean (SD)**
Age (years)	60.9 (11.2)
Time from stroke (months), range	42 (8–120)
Gait speed (m/s)	0.65 (0.20)
**Affected side**
Hip FE ROM—examination I (°)	26.25 (6.33)
Hip FE ROM—examination II (°)	31.34 (5.78)
**Unaffected side**
Hip FE ROM—examination I (°)	33.48 (5.28)
Hip FE ROM—examination II (°)	36.25 (5.45)

*N, number of subjects; SD, standard deviation; FE, flexion/extension; ROM, range of motion; °, degrees*.

### Measures

Kinematic hip data were acquired using a motion capture system (BTS SMART-DX 700, 250 Hz) equipped with six cameras, two force plates, and software, including SMART Capture, Tracker, and Analyzer functions. In line with the Davis Marker Placement system, passive markers were positioned on the participants' skin, in the following locations: the sacrum, pelvis (the posterior and anterior iliac spine), femur (lateral epicondyle, great trochanter, and in the lower one-third of the shank), fibula (lateral malleolus, lateral end of the condyle in the lower one-third of the shank), and foot (metatarsal head and heel) (29). The system was calibrated prior to each 3D assessment. During the trials which were recorded, each subject walked a distance of 10 m, and the task was repeated at least six times. The subjects were asked to walk barefoot, at their natural pace. If they excessively hesitated or lost balance during the primary trials, the procedure was repeated. For each subject, a 3D skeletal model was created. The data collected during the tests were then processed using the BTS Smart system software (Smart Tracker and Smart Analyzer). The complete ranges of hip flexion and extension in a single gait cycle were analyzed separately for the affected and unaffected sides. It was assumed that for each leg one gait cycle consisted of all the phases between two consecutive contacts of the specific foot with the ground. A recording of a single gait cycle for each leg comprised one stance phase and one swing phase. The data covering a minimum of six gait cycles performed by each subject were taken into account in the analyses. As a result, mean values of biomechanical gait parameters for the complete range of hip flexion and extension were computed, separately for the affected side and the unaffected side.

The Barthel Index (BI) enables assessment of patients' functional independence in activities of daily living. It covers 10 aspects, such as feeding, toilet use, and mobility. BI is commonly used in clinical practice in assessing performance of patients after a stroke (30, 31). It has been established that in the latter population a change of 1.85 in BI corresponds to the MCID (32). In line with the design of the current study, BI was calculated at baseline and at follow-up.

### Procedures

For the needs of the anchor-based analyses, the patients' hip ROM was measured using 3D gait assessment, at baseline (the day of the patients' admission to the rehabilitation clinic) and at follow-up (4 weeks after the first assessment). On the latter occasion, the patients were asked to report any self-perceived change in their hip ROM, observed by comparison to their condition before the therapy. Based on their self-reported perceptions, the patients were divided into three groups: the “no-change group” comprised those who reported their hip ROM was unchanged, “the MCID group” (or “positive change group”) comprised those who reported improvement, and the “negative change group” consisted of those who reported deterioration of their condition.

The distribution-based analyses took into account the no-change group only; i.e., the standard error of measurement (SEM) was computed for the patients reporting no change in their hip sagittal ROM in the period between baseline and the follow-up assessments.

A linear regression analysis was applied to examine a change in the hip sagittal ROM by comparison to the MCID in the BI.

The ROC curve approach was applied to determine the cutoff point for the change in the hip sagittal ROM, most effectively separating the “positive change group” and the “no-change group” with the ROC curve.

The therapy provided to the patients between the baseline and the follow-up continued for 150 min per day; its program comprised individual practice, including active and assisted exercise of the upper and lower limbs, balance exercise in sitting and standing position, gait reeducation, breathing exercises, and training in activities of daily living.

### Data Analysis

Statistica 13.1 (StatSoft) was used to carry out all the statistical analyses. In line with the adopted assumption, significant effects were reflected by *p* < 0.05. The comparative analyses took into account mean differences and a 95% confidence interval (CI). Four approaches were applied to calculate the indicators of change achieved in the hip FE ROM, separately for the affected and unaffected sides, and the highest value was assumed to reflect the relevant MCID. The anchor-based approach was applied to determine the first estimate for the MCID, i.e., the mean positive change in the hip FE ROM separately for the affected and unaffected sides in the MCID group (33–35). The distribution-based approach was used to identify the second estimate for the MCID; i.e., the SEM, defined as the square root of the variance of a change in the hip FE ROM, was computed for the no-change group (32, 36). Linear regression analysis was applied to determine the third estimate for the MCID, i.e., the association between the change in BI (independent variable) and the change in the hip FE ROM (dependent variable) (37, 38). The ROC curve was used to identify the fourth estimate of the MCID in the hip ROM, separately for the affected and unaffected sides, i.e., the most appropriate cutoff value for the change in the hip sagittal ROM, producing the most effective trade-off between sensitivity and specificity (39, 40).

### Sample Size

The calculation of the minimum sample size was based on the number of patients with stroke admitted annually to the rehabilitation clinic; the records showed that ~200 patients received stroke-related treatment at the facility, and 40% of those were at a chronic stage of recovery post-stroke. It was assumed that 80% of the latter patients would present a minimum gait speed exceeding 0.4 m/s and that an improvement of more than 0.06 m/s would be achieved by 80% of the patients in our group [MCID; small meaningful change for gait speed in patients after stroke; (41)]. A fraction size of 0.8 was applied, with a maximum error of 5%; as a result, a sample size of 45 patients was obtained.

## Results

As regards the anchor-based analyses, the findings showed that, at the follow-up, deterioration in the hip FE ROM was not reported by any patients as a result of which only two groups (MCID/positive change and no change) could be distinguished based on the patients' perceptions of the hip sagittal ROM. The MCID/positive change group comprised 42 patients while 8 patients were allocated to the no-change group. Table 2 presents the mean values of hip FE ROM, separately for the affected and unaffected sides in the MCID group and the no-change group.

**Table 2:** The mean hip FE ROM for the affected side and the unaffected side in the MCID group and no-change group.

**Variable—MCID group (*N* = 42)**	**Mean (SD)**
**Affected side**
Hip FE ROM—I examination (°)	25.41 (6.40)
Hip FE ROM—II examination (°)	31.21 (6.06)
**Unaffected side**
Hip FE ROM—I examination (°)	33.01 (5.32)
Hip FE ROM—II examination (°)	35.87 (5.50)
**Variable—no-change group (** ***N*** **=** **8)**	**Mean (SD)**
**Affected side**
Hip FE ROM—I examination (°)	30.66 (3.78)
Hip FE ROM—II examination (°)	31.97 (4.27)
**Unaffected side**	
Hip FE ROM—I examination (°)	35.97 (4.60)
Hip FE ROM—II examination (°)	38.24 (5.06)

*N, number of subjects; SD, standard deviation; FE, flexion/extension; ROM, range of motion; °, degrees*.

### Method 1

The anchor-based estimate was calculated taking into account the changes identified in the MCID group only. On the affected side, the mean change in hip FE ROM amounted to 5.81° and that figure was defined as the first estimate of the MCID of the hip FE ROM for the affected side. On the unaffected side, the mean change in hip FE ROM amounted to 2.86°, and that figure was defined as the first estimate of the MCID of hip FE ROM for the unaffected side (Table 3).

**Table 3:** MCID identified using four approaches for hip FE ROM—affected/unaffected side.

**Method**	**MCID (^**°**^)**	**95% CI**	
**Hip FE ROM—affected side**
Method 1	5.81	4.72	6.89
Method 2	1.56	1.03	3.18
Method 3	4.09	2.59	5.58
Method 4	2.9	–	–
**Hip FE ROM—unaffected side**
Method 1	2.86	1.54	4.18
Method 2	1.04	0.69	2.12
Method 3	0.61	−1.41	2.62
Method 4	2.6	–	–

*Method 1, anchor-based approach; Method 2, distribution-based approach; Method 3, linear regression analysis; Method 4, ROC curve; FE, flexion/extension; ROM, range of motion; °, degrees; CI, confidence interval*.

### Method 2

Distribution-based analyses were applied to the no-change group only. The SEM amounting to 1.56° was defined as the second estimate of the MCID of the hip FE ROM for the affected side. The SEM of 1.04° was defined as the second estimate of the MCID of the hip FE ROM for the unaffected side (Table 3).

### Method 3

**Figure 2:**
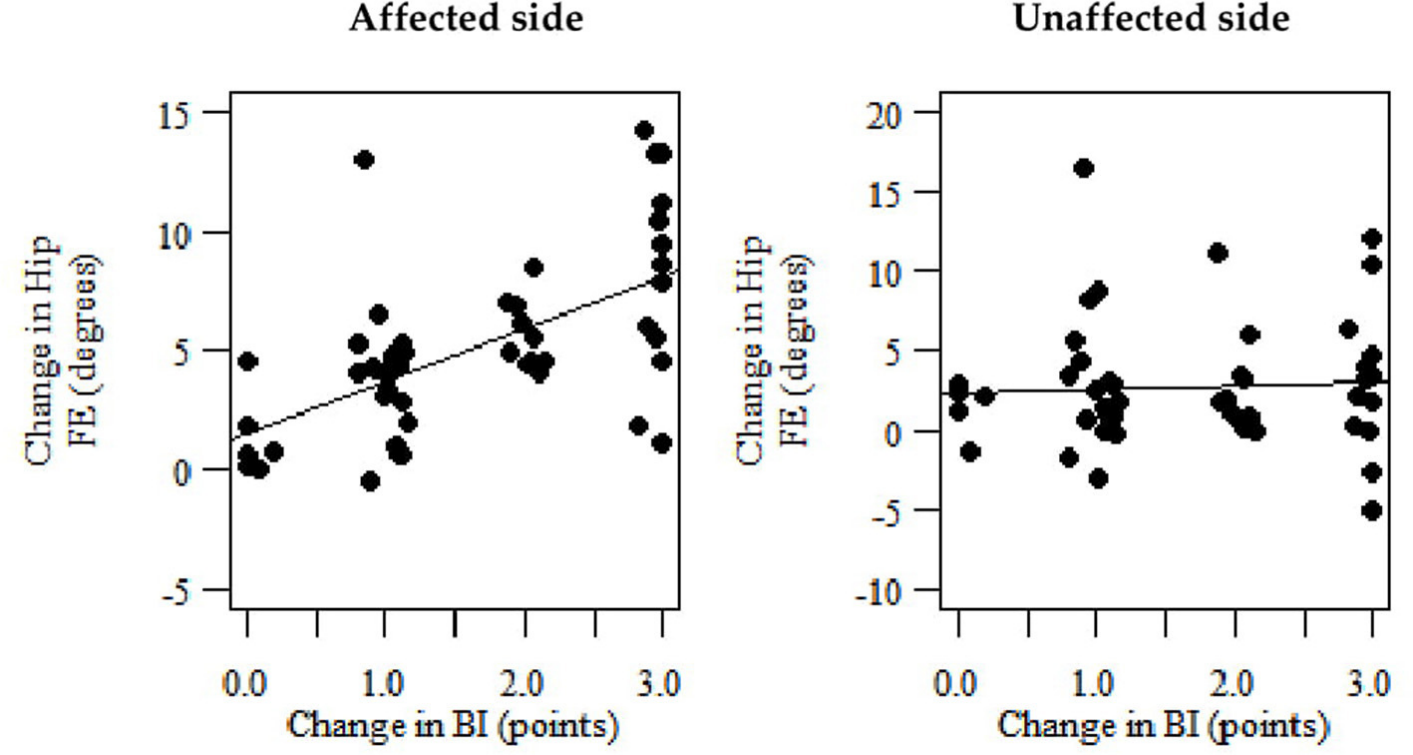
Scatterplots representing linear regression reflecting the correlation between the change in the hip FE ROM (dependent variable), separately for the affected and unaffected sides and the change in the BI (independent variable).

The slope of the regression line was 2.209 which means that a one-point change in the BI corresponded to a 2.209° change in the hip FE ROM for the affected side ROM (Figure 2). This means that a 4.09 change in the range of motion corresponds to a 1.85 change in the BI (the third MCID estimate of the hip FE ROM for the affected side) (Table 3). The slope of the regression line amounted to 0.328, i.e., a one-point change in the BI corresponds to a 0.328° change in the hip FE ROM for the unaffected side (Figure 2). This means that a 0.61 change in the range of motion corresponds to a 1.85 change in the BI (the third MCID estimate of the hip FE ROM for the unaffected side) (Table 3).

### Method 4

**Figure 3:**
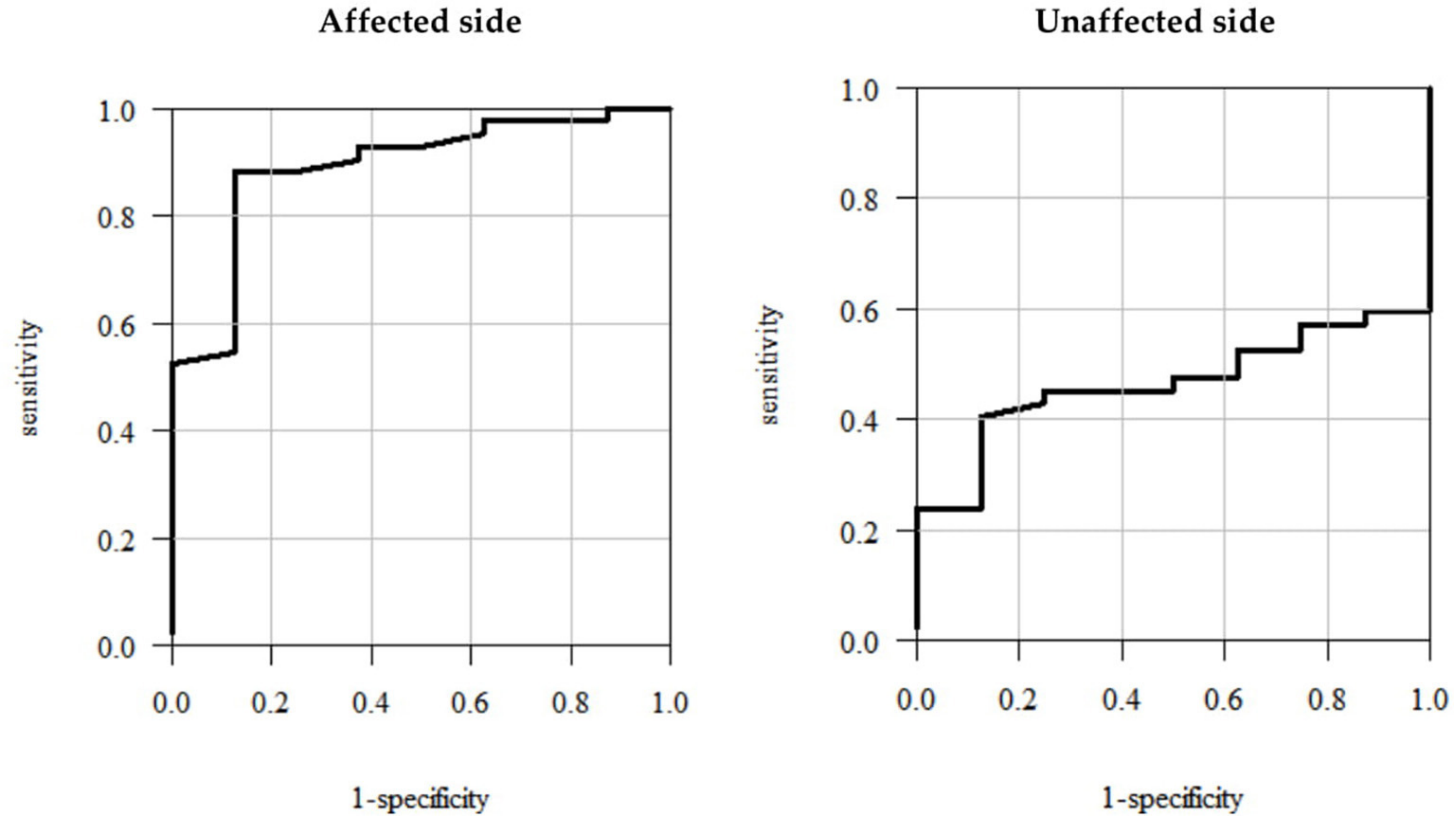
Receiver operating characteristic (ROC) curves—plots illustrating sensitivity vs. 1-specificity values for the hip FE ROM for the affected/unaffected side.

The ROC curve analyses showed that the optimum cutoff points corresponded to the values representing 2.9° and 2.6° change in the hip FE ROM for the affected and unaffected sides, respectively (Figure 3)—the fourth estimate of the MCID for the hip FE ROM for the affected/unaffected side (Table 3).

The findings related to patients with chronic stroke showed that the highest of the four MCID estimates of the hip FE ROM for the affected and unaffected sides amounted to 5.81° and 2.86°, respectively, and these values are defined as the MCID for these parameters.

## Discussion

The aim of this study was to determine MCID values for hip FE ROM for the affected and unaffected sides in patients at a chronic stage of recovery post-stroke, using four approaches: a patient anchor-based method, a distribution-based method, linear regression analysis, and specification of the ROC curve. The major findings were that the highest of the four MCID estimates of the hip FE ROM for the affected side amounted to 5.81° and for the unaffected side to 2.86°, which is the first estimate of MCID for hip FE ROM in patients with chronic stroke reported in the related literature.

Based on the literature review, it can be concluded that the effectiveness of gait reeducation can successfully be assessed using the objective evidence provided by kinematic analysis of gait (3, 7–9, 42); however, it appears that the question of the MCID for kinematic gait parameters post-stroke has generally been overlooked. To our knowledge, there is only one publication investigating the MCID for knee sagittal ROM in individuals with chronic stroke (17). The current study is a continuation of the research project which aims to determine the MCID for the kinematic parameters of the joints of the lower limbs in patients with chronic stroke. The first part of this project was designed to estimate the MCID for knee ROM in the sagittal plane for the affected and unaffected sides at a chronic stage post-stroke. We reported that in patients with chronic stroke, MCID in knee sagittal ROM on the affected side corresponded to 8.48° and on the unaffected side to 6.81 (17). Boudarham et al. (3) demonstrated that, in addition to knee impairment as a kinematic disorder in hemiplegic patients, inadequate hip function is also a predictor of walking performance. This observation provided a motivation for the present study.

The clinical relevance of these findings may be discussed by reference to intervention outcomes reported in studies focusing on similar issues. Kim et al. applied the same inclusion criteria in a study involving a group of patients similar to those in our research project; i.e., subjects who had a stroke at least 6 months earlier could walk a minimum of 10 m without assistance, had no internal medical or orthopedic problems, and understood the instructions. The researchers examined the flexion and extension ROM of the hip joint in stroke patients during obstacle-crossing tasks on the ground and underwater. They performed the respective measurements in facilities (an exercise therapy room and a therapy pool) located in a hospital building. The measurements on both the unaffected and affected sides were divided into the lead and trail sections. The authors showed significant changes (*p* < 0.05) in the flexion–extension ROM of the hip joint during the affected side trail (11.63), affected side lead (21.25), and unaffected side trail (0.61) section underwater (5). By referring these findings to the MCID identified in the present study, it is possible to observe that the change for the affected side was clinically important but for the unaffected side it was not. This may be a helpful and meaningful conclusion because it points to considerable benefits of underwater physical therapy in stroke patients with regard to affected side, and it suggests that greater attention during underwater exercise programs should be paid to the unaffected side. Likewise, Gama et al. carried out a study involving patients with chronic stroke, with similar characteristics to those presented by our group, i.e., subjects aged between 39 and 70 years (in our study 30–75 years), with time from stroke onset between 6 and 144 months (in our study 8–120 months), and with walking ability classified between levels 3 and 5 according to the Functional Ambulatory Category (in our study Functional Ambulatory Category level 3 or higher). The study aimed to assess the effectiveness of inclined treadmill training reflected by the changes in the kinematic characteristics of gait in patients with hemiparesis. The affected hip sagittal ROM showed a significant difference in time factor (6.87, *p* = 0.014) and better results after the intervention (43). If these findings are compared to the MCID identified in the current study, it is possible to infer that the change was clinically important. This would be a helpful conclusion, showing a need for treadmill training to be introduced as a regular component of gait reeducation programs designed for patients with chronic stroke. By reference to MCID values determined in the present study, it is possible to conclude that the change was not clinically important either on the affected or on the unaffected side in any of the groups. This conclusion suggests a need to continue the therapy because it seems that the short duration of the exercise program was a limitation of that study. It is likely that by sufficiently extending the duration of that specific gait reeducation program, it would have been possible to achieve longer-lasting, more sustainable, and clinically important improvements in gait.

### Limitations

This study is thought to be quite meaningful because it provides the first ever estimates of MCID for hip FE ROM, separately for the affected and unaffected sides in the chronic period post-stroke. The estimates were performed using four statistical methods, which significantly increases the likelihood that the computed values are reliable and valid. However, this study has some limitations. Firstly, estimation of the MCID for ROM of the hip joint was limited to flexion and extension. If more diverse ROM of stroke patients' hip joints (in frontal and transverse planes) were taken into account, more scientific grounds could be presented in interpreting the significance of the changes observed in the kinematic parameters of the hip; this would constitute a greater contribution toward further advancements in gait rehabilitation programs for patients at a chronic stage post-stroke. The fact that the present study focused on estimation of MCID for hip sagittal ROM only is linked with the evidence reported in the related literature, and more specifically in a robust systematic review based on 23 studies investigating the reliability of 3DGA kinematic gait measurement, and suggesting that DGA kinematic indices related to the hip and knee in the sagittal plane present the highest reliability, while the lowest reliability and highest error are frequently found in measurements of the hip and knee in the transverse plane (12). MCID for knee sagittal ROM in individuals after chronic stroke was determined in our earlier study (17). Furthermore, many researchers have pointed out that decreased sagittal ROM in the affected hip joint significantly contributes to impaired walking performance and walking speed after stroke (21–26). Given this, we decided to focus on this kinematic parameter. Secondly, our results may not be generalizable to all patients after stroke, for example those presenting more severe motor deficits, such as walking ability lower than reflected by a score of 3 according to the Functional Ambulatory Category, or motor recovery stage according to Brunnström lower than 3, and they may not be applicable to patients at acute or subacute phases of recovery post-stroke. It can be anticipated that the MCIDs determined for chronic stage post-stroke may be lower compared to those valid for the acute phase post-stroke. This may be linked with the fact that, generally the most visible recovery of neuromotor functions is achieved by stroke survivors during the first weeks following the incident (44, 45). The related effects may also be linked with patients' adaptation to the impairments and with prolonged use of compensatory gait patterns. In view of the above, these issues need to be further investigated in patients with lower motor control and in those at an acute phase post-stroke. Another problem is linked to the anchor-based method used by us; it takes into consideration changes in scores associated with specific clinical observations. It applies such external criteria as perception of the change by the medical professional or by the patient. In addition to simple estimation of difference, the “important change” construct assumes that a change has taken place and, according to the patient, clinician, or investigator, it is important (33–35). We decided to divide the participants into groups based on their self-perceived change in hip ROM because this is an external criterion commonly used by researchers (32, 34, 36, 40, 46–54), also in the case of patients at various stages and with moderate to severe hemiparesis after stroke (32, 50–54). However, stroke survivors are not always able to provide reliable information because of their sensory and/or proprioception impairments. Due to this, after acquiring information from the patients, regarding self-perceived change in the hip ROM relative to their condition before the therapy, we also carried out an assessment using another external criterion, i.e., the perception of the change by the therapist. We then compared the two assessments, which produced similar results; due to this, we decided to stick to the former option, more commonly encountered in the related literature. Certainly, we are aware of the limitations presented by this method, as this is an external criterion which may be subjective. Taking this into account, we decided to use four different statistical methods since such approach significantly increases the likelihood that the computed values are reliable and valid. Another weakness of the study is associated with the clinical scale applied. Further research is needed with regard to the Patient Reported Outcome Measures, one of these being the Global Rating of Change. This tool enables measurement of the intervention outcomes and may be helpful in determining the amount of the minimum change expected by the patients, which is of key importance in patient-centered medical services (53). Importantly, in the current study the anchor-based estimate of the MCID for hip sagittal ROM assumes a higher value than the distribution-based estimate. This means that the value corresponding to patients' self-assessed important change is higher than the measurement error, which suggests that the MCID estimate is correct (34). The latter conclusion is linked with the fact that if the situation is opposite and the patients' ratings are lower than the measurement error of a tool, it is assumed that the tool cannot reliably be applied to assess the patients' perceptions. Another limitation to the present study is the small size of the sample used in the distribution-based analyses. Further research with larger samples is needed. However, it has been reported that distribution of values in a sample may present a drawback for the distribution-based method. The size of the sample may be insufficient, leading to a lack of representativeness; apart from that, unknown factors may affect the results (35–37). It should also be pointed out that multiple factors affect the assessment of independence based on BI, which was applied in the linear regression analysis. This functional performance is not determined by independent mobility exclusively. Nevertheless, we aimed to find out whether or not a change in gait pattern associated with a change in one of its elements, i.e., hip ROM, affects patients' global functional independence in activities of daily living. A review of the related literature shows that BI is the most commonly used indicator enabling assessment of functional performance after stroke. The psychometric characteristics of BI in patients with stroke have been shown to be satisfactory (30–32). Moreover, we needed to apply a tool with an established MCID in the linear regression analyses used as a method for estimating the MCID. The latter indicator has been established for both BI and Functional Independence Measure scale (FIM), another widely used instrument (32, 39), and it has been shown that the two scales can be used interchangeably (39). Hobart et al. (55) carried out comparative analyses of the BI, the FIM, and the Functional Assessment Measure (FAM), evaluating the psychometric measures of acceptability, reliability, validity, and responsiveness of these scales, and they reported that the FIM was comparable to the BI. Similarly, Wallace et al. (56) reported that the responsiveness of the motor subscore of the BI and FIM was similar.

## Conclusions

To our knowledge, this is the first study to use a comprehensive set of statistical methods to determine the MCID for hip sagittal ROM for the affected and unaffected sides at a chronic stage of recovery post-stroke. The current study presents evidence showing that the MCID of the hip FE ROM for the affected side amounts to 5.81° and for the unaffected side to 2.86°, in patients with chronic stroke. This information is very important for clinical practice because it facilitates evaluation of progress achieved by patients as a result of interventions, enables interpretation of the changes observed in sagittal kinematic parameters of the hip, and may be used in designing gait reeducation programs.

## Data Availability Statement

The raw data supporting the conclusions of this article will be made available by the authors, without undue reservation.

## Ethics Statement

The studies involving human participants were reviewed and approved by Bioethics Commission at University of Rzeszow's Medical Faculty. The patients/participants provided their written informed consent to participate in this study.

## Author Contributions

AG: conceptualization, methodology, formal analysis, and writing—original draft preparation. MD, LP, AW-N, AT, and PK: investigation, data curation, writing—review and editing, and visualization. All the authors contributed to the article and approved the submitted version.

## Conflict of Interest

The authors declare that the research was conducted in the absence of any commercial or financial relationships that could be construed as a potential conflict of interest.

## Publisher's Note

All claims expressed in this article are solely those of the authors and do not necessarily represent those of their affiliated organizations, or those of the publisher, the editors and the reviewers. Any product that may be evaluated in this article, or claim that may be made by its manufacturer, is not guaranteed or endorsed by the publisher.
